# Supplementary material for: Electronic-reconstruction-enhanced hydrogen evolution catalysis in oxide polymorphs
Source: Nat Commun. 2019 Jul 17;10:3149. doi: 10.1038/s41467-019-11124-w (PMC6637208; doi:10.1038/s41467-019-11124-w)
Supplement: Supplementary file 1 — Supplementary information [file 41467_2019_11124_MOESM1_ESM.pdf]

Supplementary Information

Electronic-Reconstruction-Enhanced Hydrogen Evolution Catalysis in Oxide Polymorphs

Li *et al*.

This document includes:

Supplementary Figures;

Supplementary Tables;

Supplementary Notes;

Supplementary References;

Supplementary Figures

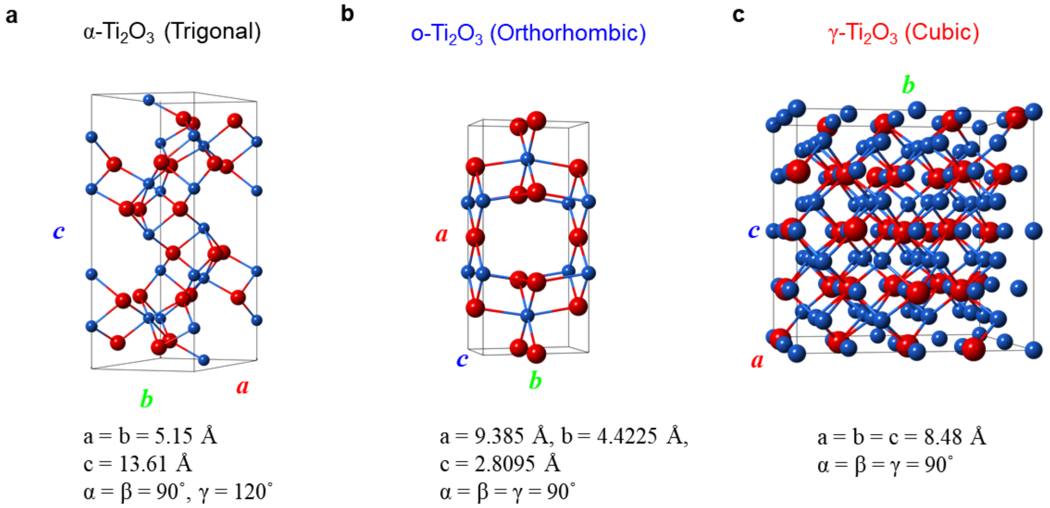

Supplementary Figure 1 | Lattice parameters for Ti_2O_3 polymorphs. a, α-Ti_2O_3, **b**, o-Ti_2O_3, **c**, γ-Ti_2O_3.

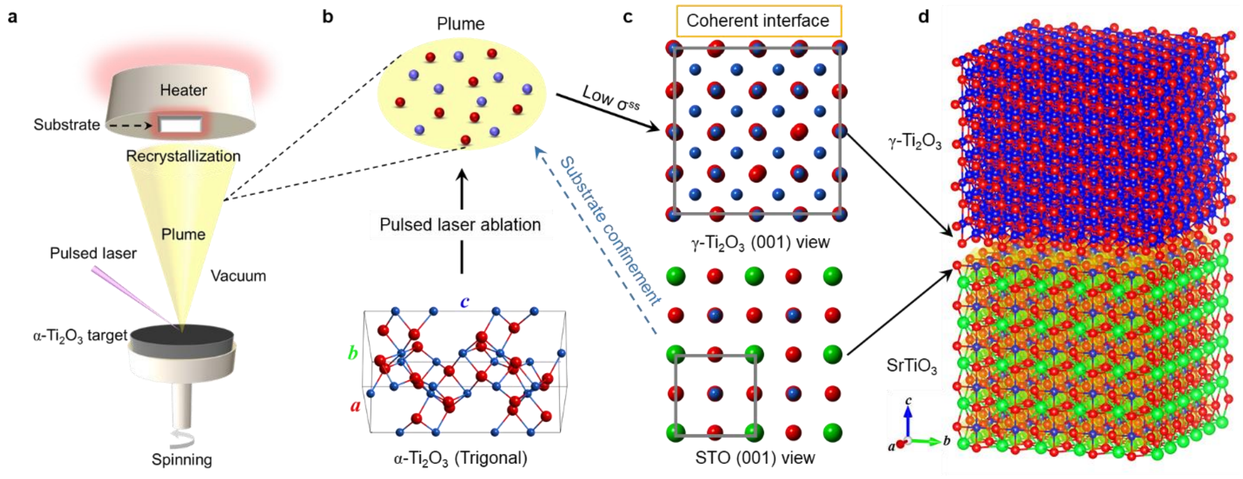

Supplementary Figure 2 │ Schematic of the epitaxial stabilization of γ-Ti_2O_3. **a** PLD process. **b** Plume formation by pulsed laser ablation. **c** Cubic/cubic coherent interface with low σ^{ss} formed between γ-Ti_2O_3 and STO due to the confinement of the substrate. **d** Epitaxial stabilization of the γ-Ti_2O_3 based on the cubic/cubic coherent interface.

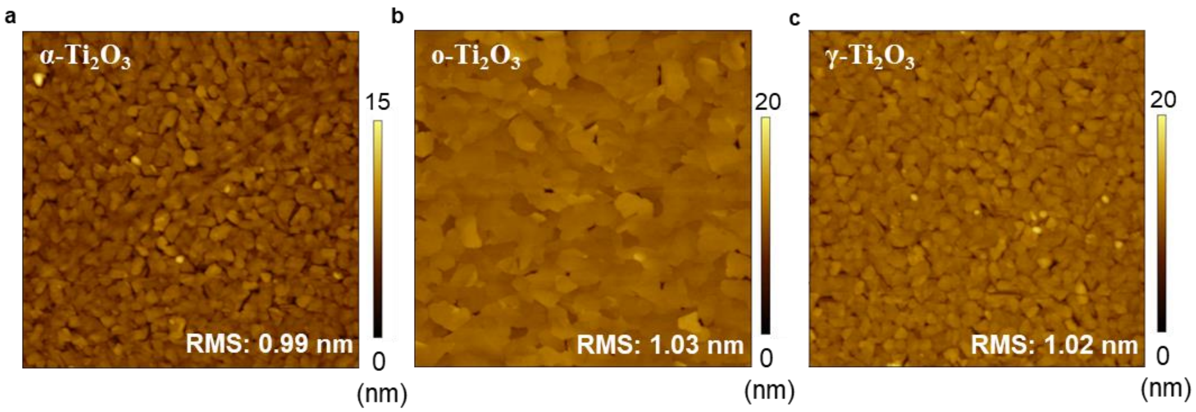

Supplementary Figure 3 | Surfaces of the Ti_2O_3 films. a-c AFM images of the α-Ti_2O_3, o-Ti_2O_3, and γ-Ti_2O_3 films, respectively. The image size is 2 × 2 μm.

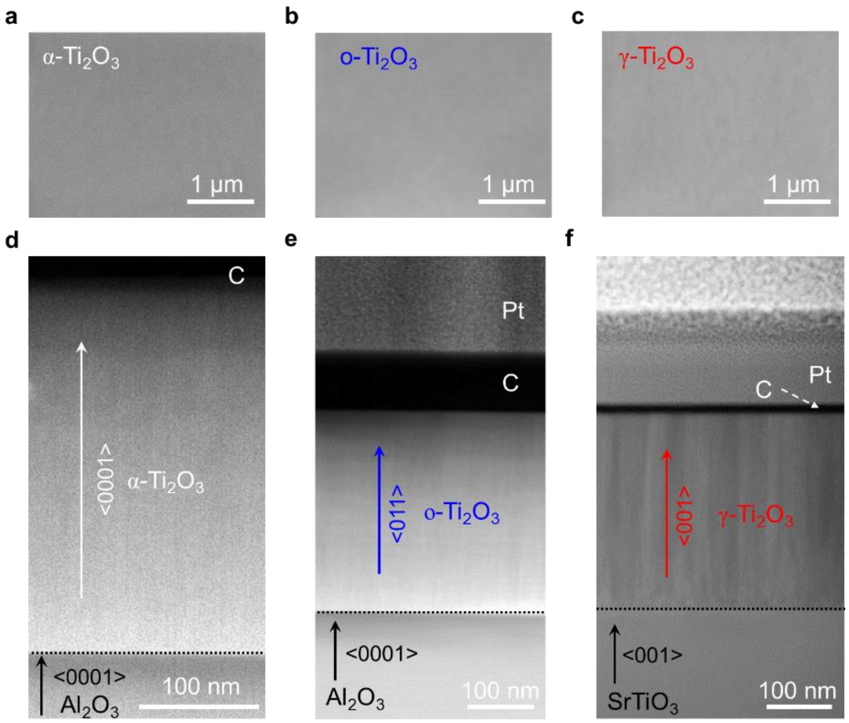

Supplementary Figure 4 | Surfaces and cross-sections of the Ti_2O_3 films characterized by electron microscopes. a-c SEM images collected from the surfaces of α-Ti_2O_3, o-Ti_2O_3, and γ-Ti_2O_3 films, respectively. **d-f** Low-resolution STEM images collected from the cross-sections of α-Ti_2O_3, o-Ti_2O_3, and γ-Ti_2O_3 films, respectively.

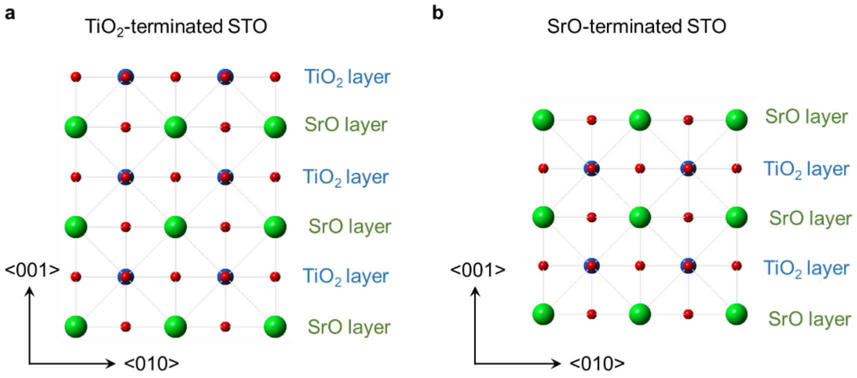

Supplementary Figure 5│STO surfaces with different terminations. **a** and **b** are schematic presentations of TiO_2-terminated and SrO-terminated STO, respectively. Along with the <001> direction, STO is made by alternative SrO layers and TiO_2 layers. Thus, the surface of STO substrates could be the TiO_2 layer-terminated or the SrO layer-terminated. Since the STO substrates used in our experiments were all etched by hydrofluoric acid and then annealed at 950 ˚C for 3 hours, the SrO layer would be removed. Then, the surface of the STO substrate is TiO_2 layer terminated. The process we used to deal with the STO substrates is a well-known and commonly utilized method to obtain the TiO_2 layer terminated STO substrates[1]. TiO_2-STO refers to "TiO_2 layer terminated STO".

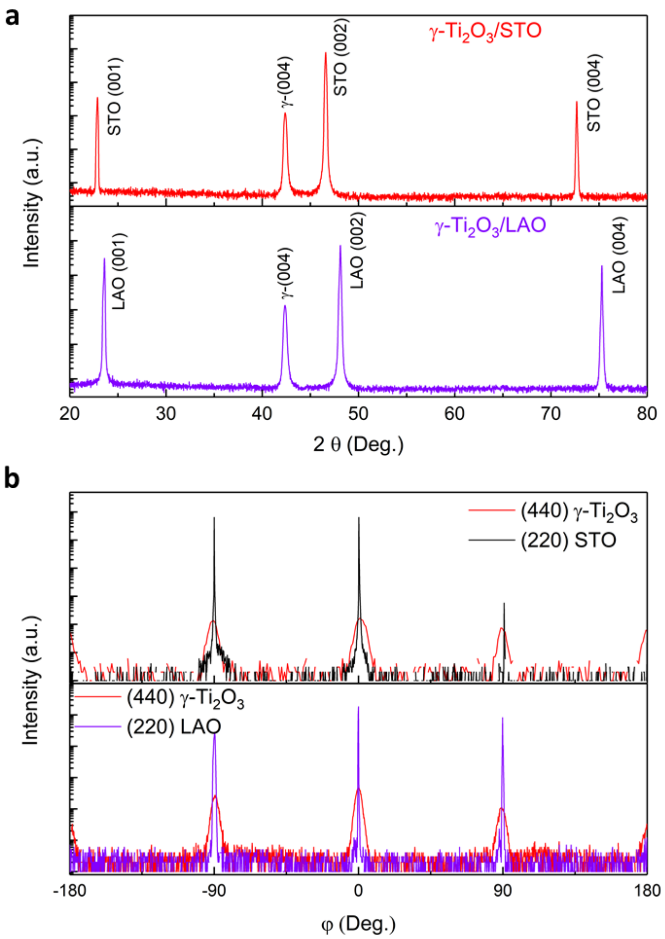

Supplementary Figure 6 | Crystal structural characterization of γ-Ti_2O_3 by XRD. a, HR-XRD patterns for γ-Ti_2O_3/STO and γ-Ti_2O_3/LAO heterostructures. **b**, Synchrotron-based in-plane φ-scans for γ-Ti_2O_3/STO and γ-Ti_2O_3/LAO heterostructures. Source data are provided as a Source Data file.

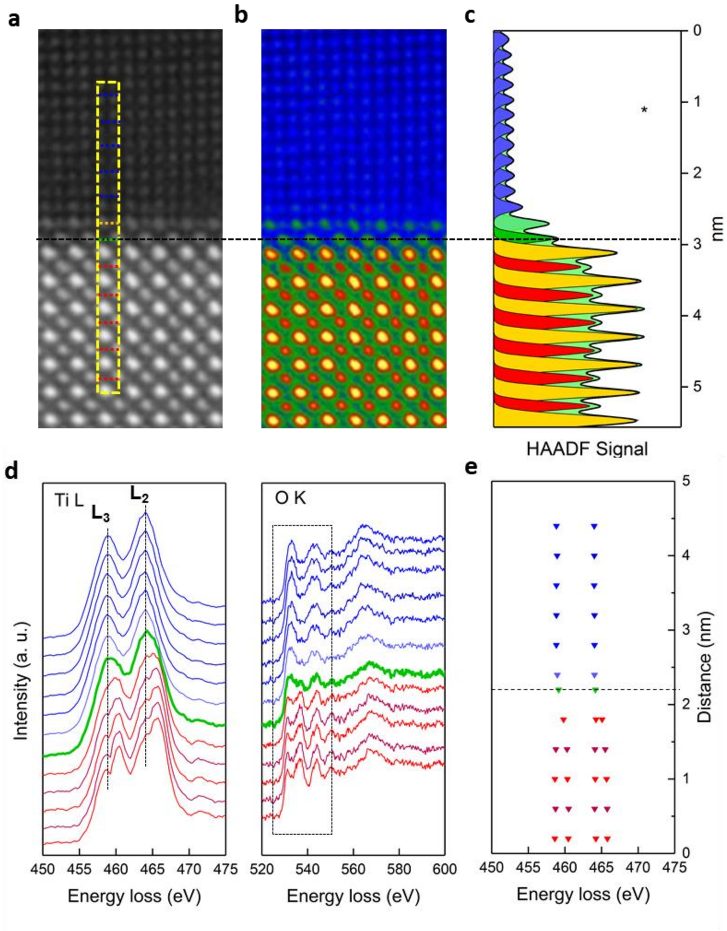

Supplementary Figure 7 | Microstructural characterizations of γ-Ti_2O_3 by HR-STEM and EELS. a and **b** are HADDF-STEM images for the γ-Ti_2O_3/STO interface, shown in the grayscale and temperature scale, respectively. The black dashed line presents the position of the TiO_2-terminated layer. **c**, HAADF signal profile obtained from **a**. **d**, EELS spectra of Ti $L_{2,3}$ and O *K*-edges obtained from the line scan across the interface shown in **a**. **e**, Peak positions of the Ti $L_{2,3}$ edge, deduced from **d**.

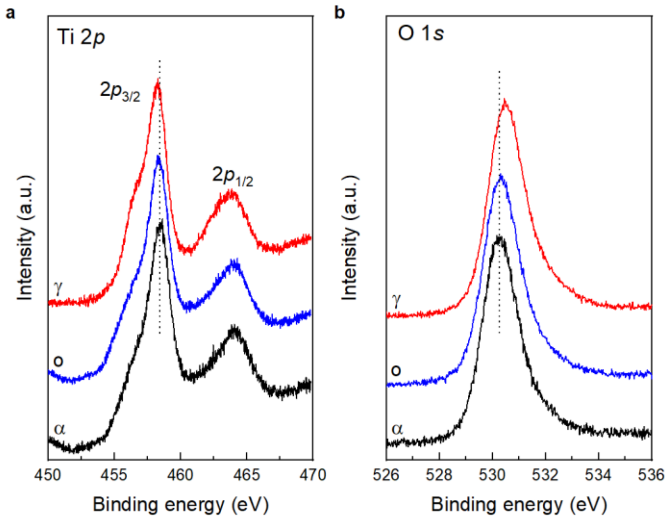

Supplementary Figure 8 | XPS. **a** Ti 2*p* and **b** O 1*s* XPS spectra collected from Ti_2O_3 polymorphs. Source data are provided as a Source Data file.

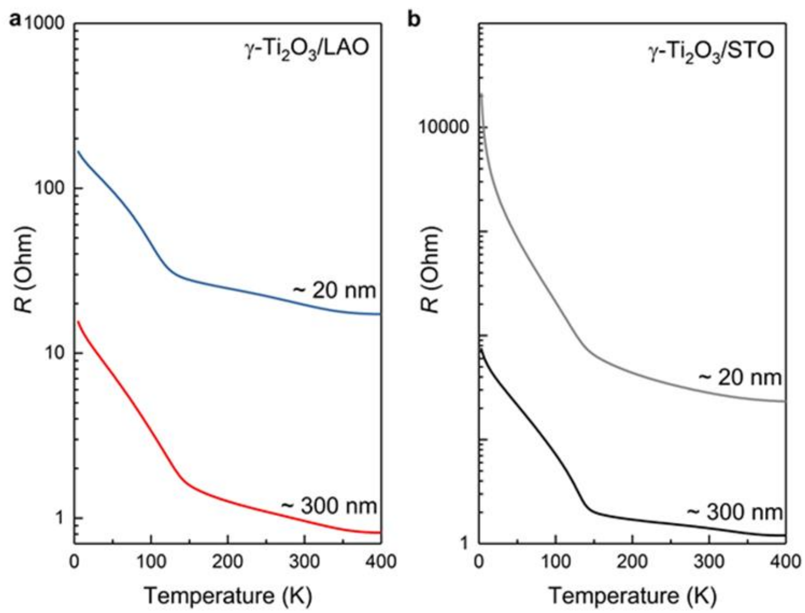

Supplementary Figure 9 | Thickness- and substrate-dependence of the electrical property of γ-Ti_2O_3. Temperature-dependent resistance of γ-Ti_2O_3 films with different thickness on **a** LAO and **b** STO substrates, respectively. Source data are provided as a Source Data file.

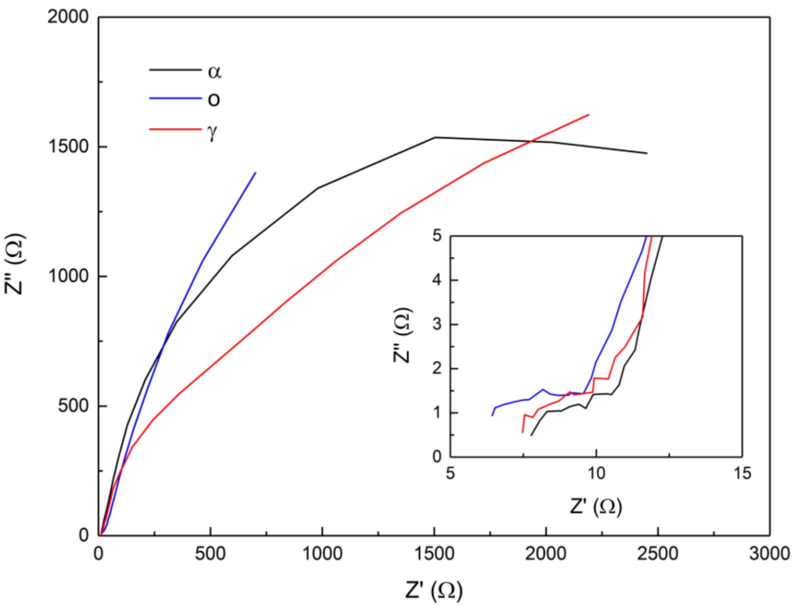

Supplementary Figure 10 | Electrochemical impedance spectroscopy. The impedance data for the Ti_2O_3 polymorphs is collected in 0.5 M H_2SO_4 and before the HER measurements. Source data are provided as a Source Data file.

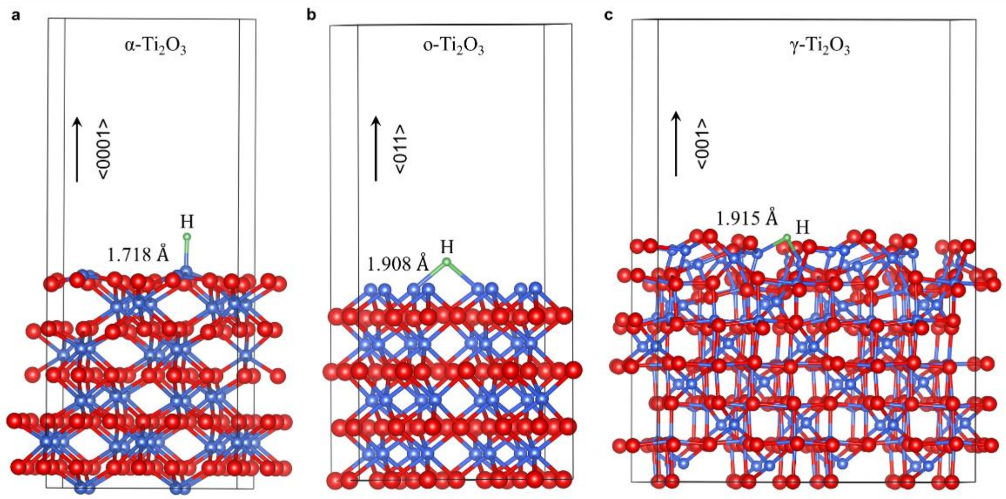

Supplementary Figure 11 │ Optimized models of H adsorbed on the surfaces of Ti_2O_3 polymorphs. **A** α-Ti_2O_3 (0001) surface. **b** o-Ti_2O_3 (011) surface. **c** γ-Ti_2O_3 (001) surface. The red, blue and green spheres represent oxygen, titanium and hydrogen atoms, respectively.

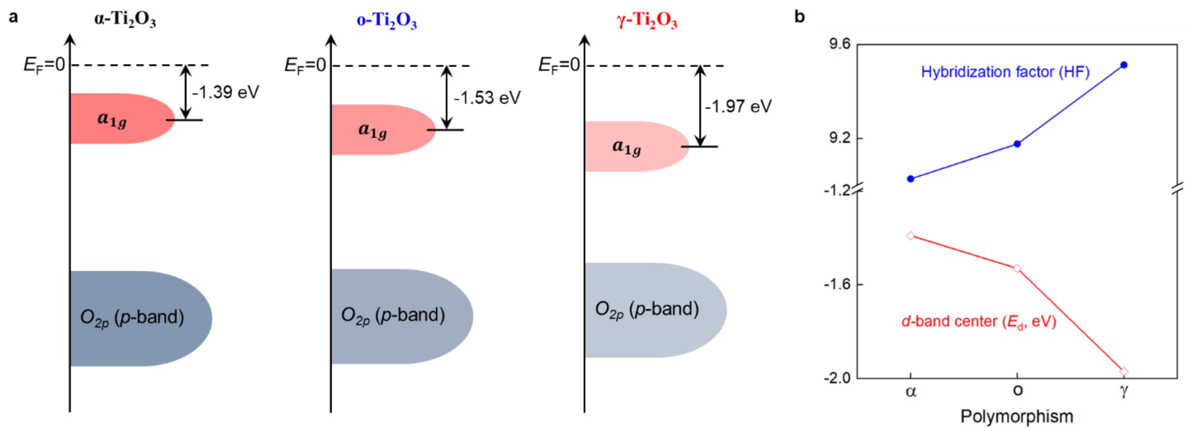

Supplementary Figure 12 | Theoretical calculation of the *d*-band center for Ti_2O_3 polymorphs. a Schematic presentation of the *d*-band center for different Ti_2O_3 polymorphs. **b** Correlation between the Ti-O hybridization and *d*-band center. Source data are provided as a Source Data file.

Supplementary Tables

Polymorphism	Ti 2$p_{3/2}$ (eV)	Ti 2$p_{1/2}$ (eV)	O 1s (eV)
α-Ti_2O_3	458.41	464.06	530.26
o-Ti_2O_3	458.35	463.95	530.30
γ-Ti_2O_3	458.20	463.73	530.48

Supplementary Table 1 | The peak positions of XPS spectra in Supplementary Figure 8.

Supplementary Note 1

It should be noted that the trigonal α-Ti_2O_3 is the thermodynamic stable phase of Ti_2O_3 and exists in bulk nature, while the cubic γ-Ti_2O_3 and orthorhombic o-Ti_2O_3 are metastable phases that have not been fabricated in the bulk-form. High-pressure high temperature measurements are usually used to study the polymorphism of materials, while the polymorphic transitions could be detected by XRD or Raman[2-5]. For Ti_2O_3, only one golden orthorhombic phase was fabricated at high temperatures and high pressures[4,6]. Thus, the formation energy of γ-Ti_2O_3 and o-Ti_2O_3 should be higher than that of the golden phase or much more energy is needed for the polymorphic transitions.

However, the epitaxial growth of oxides in the film form on the single crystal substrates is a different scenario. Pulsed laser deposition (PLD) technique was used to fabricate the Ti_2O_3 polymorphs in this work. As shown in Supplementary Figure 2a, an ultraviolet (UV: 248 nm) pulsed laser beam (pulse duration, 10-50 ns) is focused with a high energy density of ~2 J/cm^2 onto the spinning α-Ti_2O_3 target. The laser pulse is absorbed by the target, and the energy of the laser is converted to thermal energy. Rapid heating and vaporization of the target material occur at the focusing area, generating an expanding plasma plume which contains atoms, molecules, and ions, in both ground and excited states, as well as energetic electrons[7]. The atoms and ions undergo collisions in the high-density region near the target to create a highly directional expansion perpendicular to the target surface with initial velocities above 10^6 cm/s, then propagates with gradually decreased velocity to the heated substrates (6 cm away from the target). The atoms and ions will nucleate, and then grow into the epitaxial films with different orientations or phases on the surface of the substrates, depending on the nature (orientation, structure, and symmetry) of the substrates and its temperature[7]. Thus, a recrystallization process occurs on the surface of the substrates during growth. Moreover, with tunable kinetic energy of the atoms and ions inside of the plume via controlling the laser energy, the *P*-*T* phase diagram of the target material is changed during the film deposition process on the substrate's surface, making it possible to fabricate new bulk-absent metastable phases in the film form with the confinement of substrates[30].

During the epitaxial growth, the single crystalline substrates act as the seed crystal, the deposited film will lock into the same in-plane symmetry and crystallographic orientation with respect to the substrate crystal, forming the coherent or semicoherent film/substrate interface[30]. Since the energy of the coherent and semicoherent interfaces is significantly lower than that of the

noncoherent ones[8], the interfaces formed can affect the choice of the nucleus (film) crystallographic structure because the system tends to minimize the free energy to reach the equilibrium state. Thus, we can control the orientation of the films using different orientated single crystalline substrates[9], and we can also control the crystallographic structure of the film via changing the in-plane symmetry of the substrates' surface[31].

Thus, the crystallographic structure realized in the epitaxial films can be different from that of the equilibrium bulk material, which is called "epitaxial stabilization"[30,10]. From the thermodynamic point of view, epitaxial stabilization demands the change of equilibrium phase due to the epitaxial growth. In particular, the change of the pressure (P)-temperature (T) diagrams is realized for the epitaxial stabilization of metastable polymorphs[30]. We need to point out that most of the metastable phases epitaxially stabilized in the film-form could be found in the P-T phase diagram, which is the usual cases. However, there are few unusual cases that is some bulk-absent phases can only be epitaxially stabilized on the substrates in the film-form, such as body-centered cubic (bcc) Co[11], hexagonal $TbMnO_3$[31], and orthorhombic Ti_2O_3 (*Immm*)[28]. This is because the pressure-temperature induced polymorphic transition is a thermodynamic equilibrium process between different structures, while the epitaxial growth is a non-equilibrium process with the confinement of the substrates. The epitaxial stabilization of the metastable phases in the film-form is a more complicated process than the bulk phase transitions. However, it is a very fascinating method to study the emergent properties of the epitaxially stabilized metastable phases.

In this work, the trigonal α-Ti_2O_3 was grown on trigonal α-Al_2O_3 single crystalline substrates with the same (trigonal) in-plane symmetry (Figure 1e), forming the coherent film/substrate interface. For the stabilization of cubic γ-Ti_2O_3, we change the substrate from trigonal Al_2O_3 to cubic $SrTiO_3$ (STO). As shown in Supplementary Figure 2a, a plume will be generated when the pulsed laser is focused on the target (α-Ti_2O_3). Meanwhile, the crystal structure of the α-Ti_2O_3 is broken, forming the titanium and oxygen atoms or ions (Supplementary Figure 2b) inside of the plasma plume. When the atoms or ions arrived at the surface of the heated substrates (STO), the in-plane symmetry of the substrate will provide a confinement effect to drive the atoms or ions to form the (cubic γ-Ti_2O_3/cubic STO) coherent interface (Supplementary Figure 2c) on the substrate (cubic STO), minimizing the solid-solid interface energy (σ^{ss}). Based on this cubic/cubic coherent interface, the cubic γ-Ti_2O_3 is epitaxially stabilized on the cubic STO substrate (Supplementary Figure 2d).

As for the stabilization of the o-Ti_2O_3, even though the in-plane symmetry for (011) o-Ti_2O_3 and (0001) Al_2O_3 is orthorhombic and trigonal from one unit cell view (Figure 1d), which seems like a noncoherent interface. However, if we extend the unit cells, we can find a semicoherent interface between the (011) o-Ti_2O_3 and (0001) Al_2O_3. It is well known that the trigonal Al_2O_3 also has the hexagonal symmetry if we extend its *Z* number from 2 to 6. For (011) o-Ti_2O_3, the in-plane symmetry is slightly "distorted hexagonal" from the multi-unit cells view[29]. As a result, a "semicoherent" interface was formed between the (011) o-Ti_2O_3 and (0001) Al_2O_3. Thus, the orthorhombic o-Ti_2O_3 was stabilized and fabricated on the Al_2O_3 substrates in the film form. We need to point out that the deposition temperature for α-Ti_2O_3 and γ-Ti_2O_3 is 600 ˚C, while that for o-Ti_2O_3 is 900 ˚C, indicating a little bit higher energy is needed to form this "semicoherent" interface.

Supplementary Note 2

In order to determine the cubic γ-Ti_2O_3, HR-XRD and synchrotron-based in-plane φ-scans were performed. As shown in Supplementary Figure 6a, in addition to the (00*l*) peaks from the STO and LAO substrates, only (004) peak of the γ-Ti_2O_3 was observed without any impurity phase, which was consistent with that of the γ-Al_2O_3/STO heterostructure[40]. As expected, fourfold symmetry was also observed in the in-plane φ-scans (Supplementary Figure 6b) for the (440) γ-Ti_2O_3 and (220) STO or LAO, confirming the cubic-on-cubic epitaxial growth of γ-Ti_2O_3 on STO or LAO. As a consequence, the epitaxial relationship between γ-Ti_2O_3 film and STO substrate was confirmed to be (004) γ-Ti_2O_3//(002) STO and <100> γ-Ti_2O_3//<100> STO. The same scenario was shared with the γ-Ti_2O_3/LAO.

Supplementary Note 3

High resolution STEM and EELS were used to study the γ-Ti_2O_3/STO interface. Clean and shape interface was observed in Supplementary Figure 7a-c, demonstrating the good epitaxy between γ-Ti_2O_3 and STO. As mentioned in the man text, the epitaxial relationship was confirmed to be <100> γ-Ti_2O_3//<100> STO and <001> γ-Ti_2O_3//<001> STO, which was consistent with the XRD results. Furthermore, EELS line scan was performed to investigate the valence evolution of Titanium across the interface. As shown in Supplementary Figure 7d and e, four-peak feature was changed to two-peak feature across the interface, indicating the valence state of Ti was changed from 4+ to 3+[43]. Simultaneously, EELS spectra at the O *K*-edge was also varied with the evolution of Ti states, demonstrating the Ti-O hybridization was changed from STO to γ-Ti_2O_3. By now, Ti^{3+} was confirmed in the newly stabilized γ-Ti_2O_3 polymorph via both XAS (Fig. 4a) and EELS from the surface and interface, respectively. Combined with the XRD and STEM results, the new γ-Ti_2O_3, as an isomorph of γ-Al_2O_3 and γ-Fe_2O_3, was determined to stabilized on STO and LAO substrates.

Supplementary Note 4

X-ray photoelectron spectroscopy (XPS) was also performed to characterize the electronic structure and Ti-O hybridization on the Ti_2O_3 films' surfaces. As shown in Supplementary Figure 8 and Supplementary Table 1, compared to that of α-Ti_2O_3, the Ti $2p_{3/2}$ and $2p_{1/2}$ main peaks for o-Ti_2O_3 and γ-Ti_2O_3 are slightly shifted to lower binding energy, while the O 1*s* peaks are slightly shifted to higher binding energy. The binding energy of the Ti $2p_{3/2}$ main peak in γ-Ti_2O_3 is the lowest, demonstrating the strongest Ti 3*d* - O 2*p* hybridization[59], consistent with the XAS results.

Supplementary Note 5

Since the resistivity of the semiconductor can be shown as: $\rho = \frac{1}{q\,n\,\mu}$; where q is the elementary charge (1.602×10^{-19} coulombs), n and μ are concentration and mobility of the majority carriers, respectively. Thus, the resistivity is inversely proportional to the carrier concentration and mobility. That is, the resistivity will increase with the carrier concentration and mobility decrease.

Specifically, in α-Ti_2O_3 (Figure 5), the carrier concentration is decreased with decreasing temperature, which will increase the resistivity. However, the mobility is increased at lower temperatures, which will decrease the resistivity. That is, the changes of the carrier concentration and mobility with the reduced temperature have an opposite impact on its resistivity. Thus, the resistivity of α-Ti_2O_3 is increased at low temperatures as a result of the destructive effect between the carrier concentration and mobility. Similarly, in o-Ti_2O_3 (Figure 5), the carrier mobility is decreased with decreasing temperature, while the carrier concentration is anomaly increased at lower temperatures ($T<250$ K). Hence, the increase of the resistivity of o-Ti_2O_3 is also a result of the destructive effect between the carrier concentration and mobility.

However, in γ-Ti_2O_3 (Figure 5), both the carrier concentration and mobility are decreased with the temperature decrease, and they have the same impact on the resistivity, that is, they will both make the resistivity increase. So, the increase of the resistivity of γ-Ti_2O_3 is a result of the constructive effect between the carrier concentration and mobility, which results in the larger change of resistivity in γ-Ti_2O_3.

Supplementary Note 6

It should be noted that all the Ti_2O_3 samples were fabricated by the PLD technique (Supplementary Figure 2a), resulting in the dense, flat, and fully covered film/substrates heterostructures. As shown in Supplementary Figure 3, the surfaces of the films are very flat (RMS: ~1 nm). Thus, there would not be a big difference in the surface area (or specific surface area) of the Ti_2O_3 film samples.

As for the defects, in our previous work[28], those samples were deposited in very high vacuum (~3.0 × 10^{-9} Torr) in order to create some oxygen vacancies in the samples. In this work, we reduced the vacuum to around 3.0 ×10^{-6} Torr to suppress the formation of oxygen vacancies. Since the Ti_2O_3 films were deposited at high temperatures (above 600 ˚C), they could be oxidized to TiO_2 with a small amount of O_2 at the O_2 partial pressure of 5.0 × 10^{-4} Torr[28]. Depending on these experience, 3.0 × 10^{-6} Torr was confirmed to be an optimized vacuum condition to get pure-phase of Ti_2O_3 films with minimized formation of oxygen vacancies. Since we kept the vacuum at the same value (~3.0 × 10^{-6} Torr) when we fabricated all the samples in this work, the oxygen vacancies in the Ti_2O_3 polymorphs should be suppressed.

Synchrotron-based XAS was used to check the defects in the Ti_2O_3 polymorphs. As shown in Figure 4a, the Ti L_3, and L_2-edge XAS spectra of all Ti_2O_3 polymorphs are very similar, and located at ~458.6 and ~ 463.7 eV respectively, which are consistent with the bulk Ti_2O_3 samples[56], demonstrating the Ti^{3+} chemical environments in the Ti_2O_3 polymorphs. X-ray photoelectron spectroscopy (XPS) was also performed to characterize the defects on films' surfaces (Supplementary Figure 8). As expected, the Ti 2*p* and O 1*s* spectra of the Ti_2O_3 polymorphs are almost identical (with slight shifts, which results from their different Ti-O hybridization). The positions of the XPS peaks of Ti $2p_{3/2}$ and $2p_{1/2}$, located around 458.4 and 464.0 eV respectively, are consistent with those of the Ti_2O_3 bulk single crystals[12], indicating the Ti^{3+} chemical environments in those Ti_2O_3 polymorphs. By now, both XAS and XPS results confirm the Ti_2O_3 polymorphs are sharing the same Ti valence state of 3+, with minimized oxygen vacancies. Thus, with determinations of almost the same roughness (surface area) and Ti valence state in the Ti_2O_3 polymorphs, we can rule out those possible effects which may contribute to the observed polymorph-dependent HER performance.

Supplementary Note 7

Several diverse groups of materials including transition metal sulfides[13,14], selenides[15], and phosphides[16,17] have been exploited as efficient noble-metal-free HER catalysts with different benefits and drawbacks[18-20]. Recently, transition metal oxides are attracting more attention as an efficient HER catalyst[2,23,21] with compositional flexibility and environmental friendliness, whereas they were usually regarded as the HER inactive materials before, because of the unsuitable adsorption energy of H on the oxygen atoms. By now, the mechanism of the observed HER activity in oxides is still not clear. Thus, new mechanism of HER, based on the oxides' characteristics, e.g. metal-oxygen hybridization, needs to be explored for the oxide HER electrocatalysts.

Ti_2O_3 shows excellent structural flexibility[28] and tunable electronic structures with relatively high and polymorph-dependent HER performances, which provides a wonderful platform to study the HER mechanism of oxides. By investigating the electronic structures and HER in different Ti_2O_3 polymorphs via experimental and theoretical methods, strong correlation between the Ti-O hybridization and HER performance was established. Stronger hybridization of Ti 3*d* and O 2*p* orbitals lowered the *d*-band center of Ti, which weakens the H* adsorption, resulting in the enhanced HER efficiency.

We believe this conclusion could be extended to other transition metal oxide systems. Thus, based on the investigation of Ti_2O_3, a new strategy is provided to further improve the HER performance of oxides by increase the metal-oxygen hybridization, which will help for exploring more efficient oxide HER catalysts. Besides, since the samples in this work are epitaxial films with flat surfaces (Supplementary Figure 3), which is quite different from those of the other groups of materials discussed above, the HER active area of the Ti_2O_3 (flat films) is actually limited. Thus, we believe the HER activity of the Ti_2O_3 (films) could be further enhanced by increasing the surface area via nanostructure-array fabrications.

Supplementary References

1. S. A. Chambers, *et al*, Unintentional F doping of $SrTiO_3$(001) etched in HF acid-structure and electronic properties. *Surf. Sci.* **606**, 554 (2012).

2. Ono S, et al. High-pressure phase transition of hematite, Fe_2O_3. *J. Phys. Chem. Solids* **65**, 1527 (2004).

3. Ono S, *et al*. *In situ* X-ray observation of phase transformation in Fe_2O_3 at high pressures and high temperatures. *J. Phys. Chem. Solids* **66**, 1714 (2005).

4. V. O. Sergey, *et al*. Structural stability of a golden semiconducting orthorhombic polymorph of Ti_2O_3 under high pressures and high temperatures. *J. Phys. Condens. Matter*. **22**, 375402 (2010).

5. S. H. Shim, *et al*. Raman spectroscopy and x-ray diffraction of phase transitions in Cr_2O_3 to 61 GPa. *Phys. Rev. B* **69**, 144107 (2004).

6. V. O. Sergey, *et al*. High-pressure behavior of structural, optical, and electronic transport properties of the golden Th_2S_3-type Ti_2O_3. *Phys. Rev. B* **88**, 184106 (2013).

7. D. H. Lowndes, *et al*. Synthesis of novel thin-film materials by pulsed laser deposition. *Science* **273**, 898 (1996).

8. A. P. Sutton; R. W. Balluffi. *Interfaces in crystalline materials*; Calendron Press: Oxford, 1995.

9. Er-Jia Guo, *et al*. Orientation control of interfacial magnetism at $La_{0.67}Sr_{0.33}MnO_3/SrTiO_3$ interfaces. *ACS Appl. Mater. Interfaces* **9**, 19307−19312 (2017).

10. A. Kaul, *et al*. Epitaxial stabilization - A tool for synthesis of new thin film oxide materials. *J. Cryst. Growth* **275**, e2445 (2005).

11. G. A. Prinz, Stabilization of bcc Co via epitaxial growth on GaAs. *Phys. Rev. Lett.* **54**, 1051 (1985).

12. R. L. Kurtz, *et al*. Comparison of Ti 2p core-level peaks from TiO_2, Ti_2O_3, and Ti metal, by XPS. *Surf. Sci. Spectra* **5**, 179 (1998).

13. D. Voiry, *et al*. Enhanced catalytic activity in strained chemically exfoliated WS_2 nanosheets for hydrogen evolution. *Nat. Mater.* **12**, 850 (2013).

14. D. Voiry, *et al*. Conducting MoS_2 nanosheets as catalysts for hydrogen evolution reaction. *Nano Lett*. **13**, 6222 (2013).

15. D. Kong, *et al*. $CoSe_2$ nanoparticles grown on carbon fiber paper: an efficient and stable electrocatalyst for hydrogen evolution reaction. *J. AM. Chem. Soc.* **136**, 4897 (2014).

16. H. Yang, *et al*. Urchin-like CoP nanocrystals as hydrogen evolution reaction and oxygen reduction reaction dual-electrocatalyst with superior stability. *Nano Lett.* **15**, 7616 (2015).

17. P. Xiao, *et al*. A review of phosphide - based materials for electrocatalytic hydrogen evolution. *Adv. Energy Mater.* **5**, 1500985 (2015).

18. Y. Yan, *et al*. A review on noble-metal-free bifunctional heterogeneous catalysts for overall electrochemical water splitting. *J. Mater. Chem. A* **4**, 17587 (2016).

19. S. Anantharaj, *et al*. Recent trends and perspectives in electrochemical water splitting with an emphasis on sulfide, selenide, and phosphide catalysts of Fe, Co, and Ni: A review. *ACS Catal.* **6**, 8069 (2016).

20. D.H. Ha, *et al*. Activity and stability of cobalt phosphides for hydrogen evolution upon water splitting. *Nano Energy* **29**, 37-45 (2016).

21. Y. Zhu, *et al*. Unusual synergistic effect in layered Ruddlesden−Popper oxide enables ultrafast hydrogen evolution. *Nat. Commun.* **10**, 149 (2019).
